# Benefits and Challenges of Pre-clustered Network-Based Pathway Analysis

**DOI:** 10.3389/fgene.2022.855766

**Published:** 2022-05-10

**Authors:** Miguel Castresana-Aguirre, Dimitri Guala, Erik L. L. Sonnhammer

**Affiliations:** Department of Biochemistry and Biophysics, Science for Life Laboratory, Stockholm University, Stockholm, Sweden

**Keywords:** functional association networks, network clustering, biological mechanisms, pathway enrichment analysis, sensitivity increase

## Abstract

Functional analysis of gene sets derived from experiments is typically done by pathway annotation. Although many algorithms exist for analyzing the association between a gene set and a pathway, an issue which is generally ignored is that gene sets often represent multiple pathways. In such cases an association to a pathway is weakened by the presence of genes associated with other pathways. A way to counteract this is to cluster the gene set into more homogenous parts before performing pathway analysis on each module. We explored whether network-based pre-clustering of a query gene set can improve pathway analysis. The methods MCL, Infomap, and MGclus were used to cluster the gene set projected onto the FunCoup network. We characterized how well these methods are able to detect individual pathways in multi-pathway gene sets, and applied each of the clustering methods in combination with four pathway analysis methods: Gene Enrichment Analysis, BinoX, NEAT, and ANUBIX. Using benchmarks constructed from the KEGG pathway database we found that clustering can be beneficial by increasing the sensitivity of pathway analysis methods and by providing deeper insights of biological mechanisms related to the phenotype under study. However, keeping a high specificity is a challenge. For ANUBIX, clustering caused a minor loss of specificity, while for BinoX and NEAT it caused an unacceptable loss of specificity. GEA had very low sensitivity both before and after clustering. The choice of clustering method only had a minor effect on the results. We show examples of this approach and conclude that clustering can improve overall pathway annotation performance, but should only be used if the used enrichment method has a low false positive rate.

## Introduction

The advance in high throughput experiments has led to a huge increase in the data available for understanding biological function. However, extracting function from high-throughput experiments is often not straightforward since genes and proteins are involved in many different biological mechanisms and pathways. The quest for biological insight from high-throughput experiments has therefore prompted the invention of a large number of pathway enrichment analysis tools.

The most recent family of pathway analysis methods are the network-based tools, such as EnrichNet (Glaab et al., 2012), NEAT (Signorelli et al., 2016), NEArender (Jeggari and Alexeyenko, 2017), BinoX (Ogris et al., 2017), and ANUBIX (Castresana-Aguirre and Sonnhammer, 2020). These methods require a functional association network, such as FunCoup (Persson et al., 2021) or STRING (Szklarczyk et al., 2021), where different types of data describing relationships between genes and/or proteins, are integrated to infer functional associations between genes. Using enrichment of network links, instead of overlap between gene sets, substantially improves the chances of detecting a relationship, as networks provide much more information (Ogris et al., 2017). Statistical significance of network-based pathway analysis methods is assessed based on the network crosstalk, i.e., links connecting the studied gene set and the pathway of interest. Methods such as BinoX rely on network randomization to obtain a null distribution, which is fit to a binomial distribution to compute the expected crosstalk. NEAT and NEArender compute the expected crosstalk based on the node degree of the query, the pathway and the network, with the difference that NEAT fits a hypergeometric distribution and NEArender a chi-square distribution, but their results are very similar. ANUBIX randomly samples gene sets of the same size as the original query set and fits the expected crosstalk to a beta-binomial distribution. While all these methods except ANUBIX have been shown to suffer from high false positive rates when testing random gene sets for enrichment (Castresana-Aguirre and Sonnhammer, 2020), we here included BinoX and NEAT, together with ANUBIX to study how clustering affects different methods.

**FIGURE 1:**
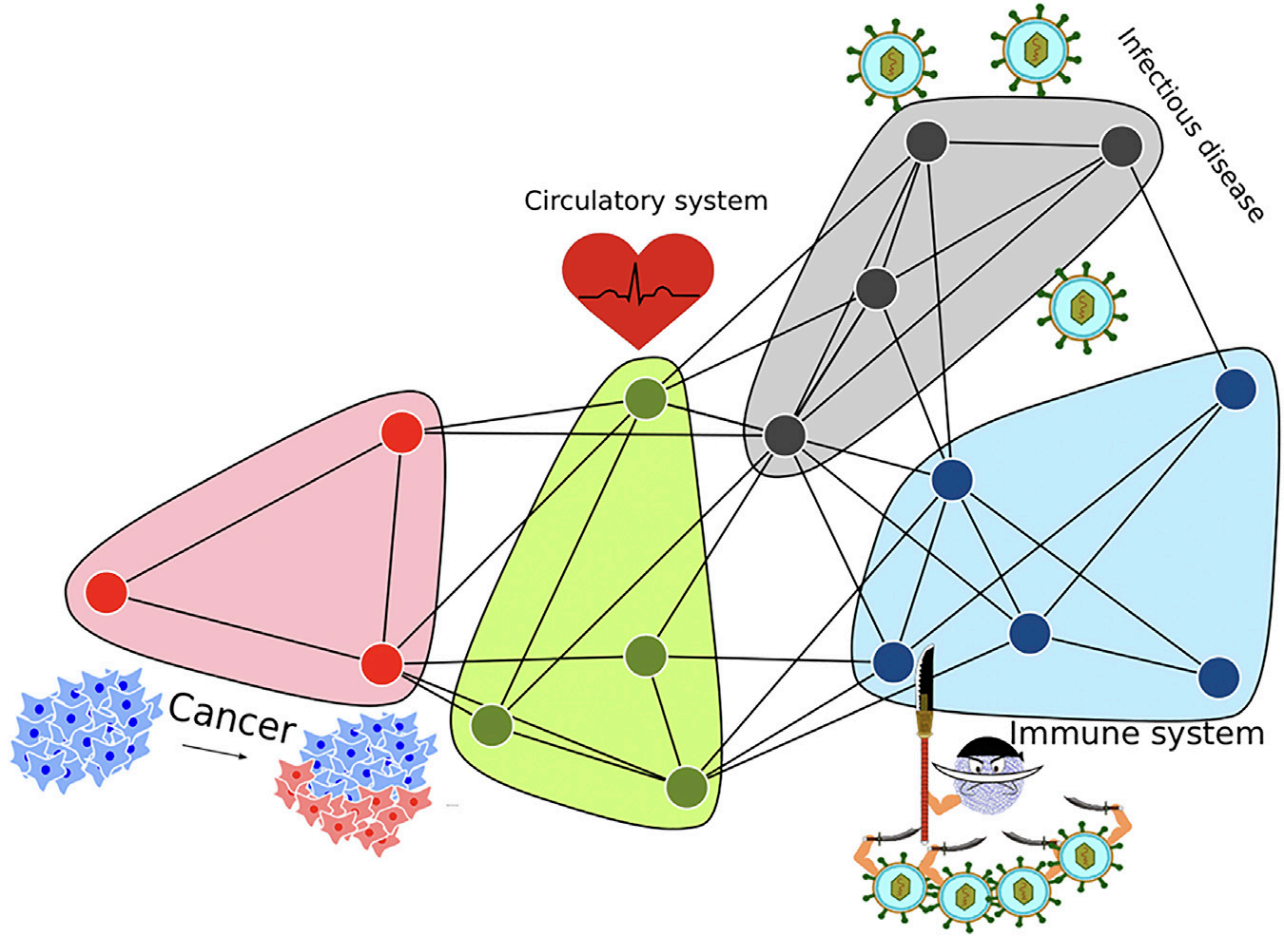
Gene sets derived from experiments are often complex with multiple affected pathways. This illustration shows genes that belong to 4 pathways that are functionally distinct. The mixture of pathways may complicate the pathway enrichment analysis, especially for smaller pathways. By separating gene clusters prior to pathway analysis, a clearer picture of the pathway enrichment can be obtained.

Network-based methods provide the highest sensitivity of all the pathway enrichment families (Ogris et al., 2017; Castresana-Aguirre and Sonnhammer, 2020). However, experimental gene sets are often complex with multiple affected pathways, which increases noise and leads to decreased sensitivity. An example of this would be a gene set consisting of four functional modules where each one is enriched for a specific pathway (Figure 1). A pathway analysis method would struggle to detect each module’s pathway association if the genes belonging to each module is only a small fraction of all genes in the gene set. Additionally, the studied gene set could contain noise in the form of other genes not related to the main phenotypes of the gene set, which could cause false negatives, impacting the sensitivity of pathway analysis.

Due to the ubiquitous use of pathway analysis methods and reliance on their output to interpret results from diverse and important fields of research such as drug development (Jhamb et al., 2019), biomarker discovery (Chen et al., 2017) and patient diagnosis (Lu et al., 2019), it is important to ensure that these methods can cope well with complex gene sets.

One way to achieve this is to reduce the mentioned complexity by separating the mix of affected pathways. Clustering is a technique that has been used to lower complexity of data by grouping similar entities in various fields, such as pattern recognition (Baraldi and Blonda, 1999; Chen and Huang, 2003), image analysis (Chen et al., 2015; Dhanachandra et al., 2015), and analysis of biological interaction networks (Ideker et al., 2002; Opresko et al., 2004; Mitra et al., 2013). In the field of pathway analysis, clustering is used in PathFindR (Ulgen et al., 2019) and GScluster (Yoon et al., 2019) to find subnetworks or modules in a gene set mapped to a protein-protein interaction (PPI) network, followed by gene overlap based pathway analysis. However, neither of these tools have evaluated the combination of clustering with state-of-the-art pathway analysis methods, nor have they compared the performance of used methods with and without clustering.

The approach we take here is applying clustering to decrease complexity of the gene sets, and then apply state-of-the-art network-based pathway enrichment methods. We first investigated whether top-performing clustering methods such as MCL, Infomap, and MGclus are able to extract single pathways from pathway mixtures. The performance of clustering in combination with the network-based pathway analysis methods BinoX, NEAT, and ANUBIX, as well as classical overlap-based Gene Enrichment Analysis (GEA), was evaluated using a benchmark constructed based on the KEGG pathway database.

## Materials and Methods

Clustering is a way to group objects into different communities, where the objects within each community are more similar to each other than to objects in the other communities (Malliaros and Vazirgiannis, 2013). When clustering is used in the context of a network it involves grouping nodes with high intra-module density, i.e., that are highly connected within a network neighborhood and less connected to the nodes outside said community. There are different types of clustering, e.g., connectivity clustering, centroid clustering, density clustering, distribution clustering, network-based clustering, etc. (Emmons et al., 2016). In our study we focus on network-based clustering, since we are mapping a query gene set onto a network. Since the purpose of this study is not to benchmark the clustering methods themselves, we decided to pick three methods. These methods are MGclus, which has been shown to work well with the FunCoup network (Frings et al., 2013), Infomap (Rosvall and Bergstrom, 2008), and MCL (Van Dongen, 2008), due to their superior performances compared to other methods (Lancichinetti and Fortunato, 2009; Shemirani et al., 2021).

### Clustering Methods

MGclus defines modules based on the intra- versus inter-connectivity in a module and considers shared neighbors of nodes as evidence that they belong to the same module.

Both Infomap and MCL extract modules using random walks on the underlying network. MCL performs an iterative random walk along the edges of the network to discover where the flow tends to gather. These iterative random walks are calculated using Markov chains, where the transition probability matrix changes in each run. Infomap finds the optimal set of modules that minimizes the information required to describe a random walk through a network. The description is in two levels, coding for nodes and modules (Rosvall et al., 2009). All clustering algorithms were used with their standard configurations.

### Pathway Analysis Tools

GEA is an overlap-based method that tests if the overlap between two sets of genes is higher than would be expected by chance. Statistical significance is assessed using a modified Fisher’s exact test where random overlap is modeled from random samples of pairs of gene sets. This test is a conservative variation of Fisher’s exact test, where 1 is subtracted from the observed overlap, as in DAVID’s (Huang et al., 2009) EASE score. This means that GEA cannot determine statistical significance of overlaps smaller than 2 nodes.

BinoX assumes that the random crosstalk between two gene sets in the network is distributed according to the binomial distribution. It therefore randomizes the network and computes a distribution of pairs of randomly drawn gene sets to estimate the parameters of a binomially distributed random crosstalk. These parameters are used to determine the expected crosstalk. BinoX can assess whether a pathway is enriched or depleted for the studied gene set. A depleted pathway means that the gene set has fewer links to the pathway than expected by chance.

NEAT and NEArender use slightly different assumptions about the distribution of random crosstalk in the network. NEAT assumes a hypergeometric distribution of crosstalk while NEArender assumes a chi-square distribution. Therefore, instead of testing the observed crosstalk between the studied gene set and a pathway of interest using a sampled random distribution, they rely on the hypergeometric and chi-square test respectively to assess statistical significance. However, both methods compute the expected crosstalk in the same way, taking into account the degree of the gene set, the pathway and the network. Both methods can compute enrichment and depletion. Since NEAT and NEArender show very similar results, we only selected one of them (NEAT) for our benchmark.

ANUBIX is a novel network-based method that computes the enrichment of a gene set for a pathway of interest based on the network crosstalk. The observed crosstalk is assessed for statistical significance using a model of the null distribution of the random crosstalk in the network. This null distribution is modeled by drawing random samples of gene sets, of the same size as the studied gene set, from the genome, calculating their crosstalk with the pathway of interest and fitting the parameters of a beta-binomial distribution for the distribution of the random crosstalk. The procedure can be applied to one or multiple pathways of interest. The statistical significance of the observed crosstalk is only assessed for enrichment, where the observed crosstalk is larger than would be expected by chance.

### Null Model Modification of ANUBIX

To generate a null distribution of random crosstalk, ANUBIX samples gene sets from the genome, at random. The assumptions behind this null distribution may be weak when the gene sets under study contain genes not present in the used functional association network or have node degrees that deviate from the expected degrees when drawing random genes. To make the underlying null model more accurate we used degree-aware node sampling (McCormack et al., 2013) to construct the underlying distribution. We achieved this by first grouping all network nodes into bins, one per degree if more than 100 nodes exist for a given degree, or bins representing a range of degrees if this was needed to obtain at least 100 nodes in the bin. Sampling to produce random gene sets was done by randomly selecting nodes from bins with the same degree as the nodes in the query set.

To assess the improvement of this modification, we generated 100 random gene sets by sampling from the whole genome and another 100 random gene sets by sampling from the subset of genes present in all Chemical and Genetic interaction (CGP) gene sets in the Molecular Signatures Database (MSigDB) (Liberzon et al., 2011). Sampling was done such that the gene frequencies in the MSigDB gene sets were preserved. The size of the gene sets was fixed to 50 genes, which was the median size of all the gene sets in MSigDB.

### Functional Association Network

Network-based pathway enrichment methods require a protein interaction network. In our study we used FunCoup, which is one of the most comprehensive functional association networks of genes/proteins available. FunCoup infers functional associations between genes by integrating different types of evidence using a redundancy-weighted naïve Bayesian approach, combined with orthology transfer. FunCoup’s high coverage comes from the number and variety of different evidence types used, such as: mRNA and protein co-expression, co-evolution based on phylogenetic profile similarity, Protein-Protein and domain-domain interactions, sub-cellular co-localization, co-regulation via miRNA and transcription factors, as well as genetic interaction.

For this study, we used the *Homo sapiens* FunCoup 5 network. To avoid noise, we used the default link confidence cutoff of 0.8 resulting in a network of 612,276 links and 12,890 genes.

### Pathway Database

For this study we use the 313 *H. sapiens* pathways from the Kyoto Encyclopedia of Genes and Genomes (KEGG) (v.96.0) (Kanehisa et al., 2016).

## Benchmarks

### Pathway Recovery for Each Clustering Method

Performance of clustering algorithms may vary depending on the properties of the network they are applied to, so we constructed a simple benchmark to assess this. We generated 100 gene sets by merging different KEGG pathways that had shared links, three pathways at a time. Then we applied the different clustering methods to these gene sets to produce modules. Each module was assigned to the pathway with the highest overlap, and the Jaccard index between the sets of assigned and true pathways was computed for each method. The Jaccard index distributions of the clustering methods were compared using Kruskal-Wallis and Wilcoxon tests.

### True Positive Benchmark

KEGG pathways were bisected into two parts with similar number of nodes and total node degree. The overlap between the bisected parts was emulated based on the median overlap between gene sets in the MSigDB database and KEGG pathways. KEGG pathways were ordered by size and grouped into seven bins with an equal (or as equal as possible) number of pathways in each bin. We then sampled one pathway from each bin at random and merged them into a unique gene set. To decide how many pathways to join, we performed a pathway analysis study of Chemical and Genetic interaction (CGP) MSigDB gene sets against KEGG pathways using the null model modified ANUBIX. To keep a reasonable gene set size, and to avoid merging too many pathways, we used Bonferroni correction (Abdi, 2007) and a family-wise error rate (FWER) of 5% as a cutoff. This resulted in a median number of significantly enriched pathways of seven per gene set. We therefore chose to join seven pathways for the construction of the multi-pathway gene sets. Since our sampling was constrained by the binning procedure, to avoid having too much overlap between the constructed gene sets, but still retain a statically usable number of gene sets we generated 100 gene sets and ran pathway enrichment against the other parts of the bisected pathways. Since each gene set was constructed from seven different pathways and we were aiming to recover the other half of each of those pathways, we could at most have 700 true gene set-pathway associations or True Positives (TPs).

### False Positive Benchmark

For the false positive (FP) benchmark we generated 100 random gene sets of the average size of the true positive gene sets, 280 genes. The generated gene sets were tested for enrichment against the true KEGG pathways. Considering their randomness, we did not expect to find any enriched pathways.

### Performance Measures

**FIGURE 2:**
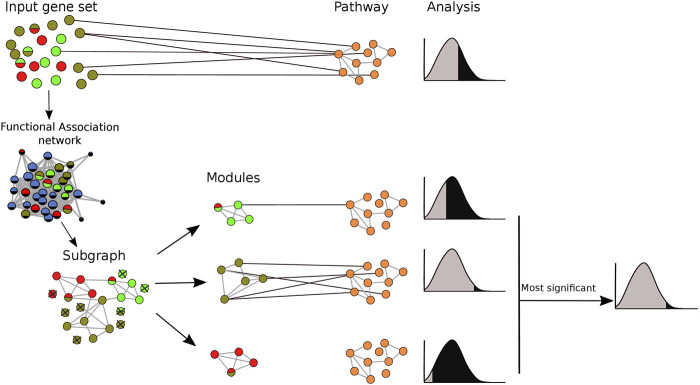
Integrating clustering methods into pathway enrichment analysis. The input gene set was mapped onto an association network and clustering algorithms were applied. Based on this, the gene set was divided into several modules, and pathway enrichment analysis was run for each of the modules separately, keeping only the most significant result for each module-pathway pair. Pathway enrichment was also run in the original gene set for comparison purposes. Each node represents a gene. Different colors refer to membership in different modules or pathways. A multicolored circle indicates more than one membership.

Both the true positive and false positive benchmarks were applied with and without clustering of gene sets prior to pathway analysis. When clustering was applied, pathway enrichment was tested individually for each identified module. The pathways with the lowest *p*-value for each module were merged into a single list. The performance of each method was assessed by Receiver Operator Characteristics (ROC) curves (Bradley, 1997). For our analysis, we select only the pairs that were statistically significantly (FDR < 0.05) enriched after adjusting *p*-values using the Benjamini-Hochberg procedure (Benjamini and Hochberg, 1995). The pipeline of the clustering implementation in pathway enrichment analysis is shown in Figure 2.

### Adaptive Module Size Filtering

Applying clustering to the query gene sets increases the sensitivity of the underlying analysis. However, this often comes with an increase in false positives, mainly stemming from small modules. To control for this, we devised a filtering approach for small modules prior to the pathway enrichment analysis. To calibrate it, we generated 100 random gene sets for a range of sizes between 50 and 600 genes, increasing the size by 50 genes, and ran the clustered pathway enrichment pipeline against KEGG pathways. At FDR < 0.05, we studied which minimum module size cutoff was necessary to keep the FPR below 5%. With the selected range of gene set sizes, we observed that the required module size cutoff increased linearly with the query gene set size (Supplementary Figure S1), suggesting that the cutoff should be adapted to different gene set sizes. This approach only works well for methods that already control the FPR well prior to clustering, here yielding good results only for ANUBIX. The adaptive module size filtering ensures an FPR level matching the set FDR level in ANUBIX when filtering out modules whose size is below 2% of the query gene set size, hence this filter was applied to ANUBIX here. For BinoX and NEAT this was however not possible to achieve without a massive loss of sensitivity, hence the filter could not be applied to them.

### Clustered vs. Non-Clustered MSigDB Gene Sets Analysis

We ran pathway enrichment analysis against KEGG pathways for all the CGP MSigDB gene sets in two different scenarios, with and without pre-clustering the gene sets. To showcase that different gene sets are a mixture of different pathway or pathway families, for each MSigDB gene set, we studied how often a certain pathway subclass, as defined by KEGG, was targeted by the same gene set module. The KEGG database classifies pathways into 6 classes and 42 subclasses. The overlap in significantly enriched pathways between (A) with pre-clustering and (B) without pre-clustering was computed using the Jaccard Index as described in Eq. 1:

$$J\left(A,B\right)=\frac{\left|A\cap B\right|}{\left|A\cup B\right|}$$ (1)

## Results

**FIGURE 3:**
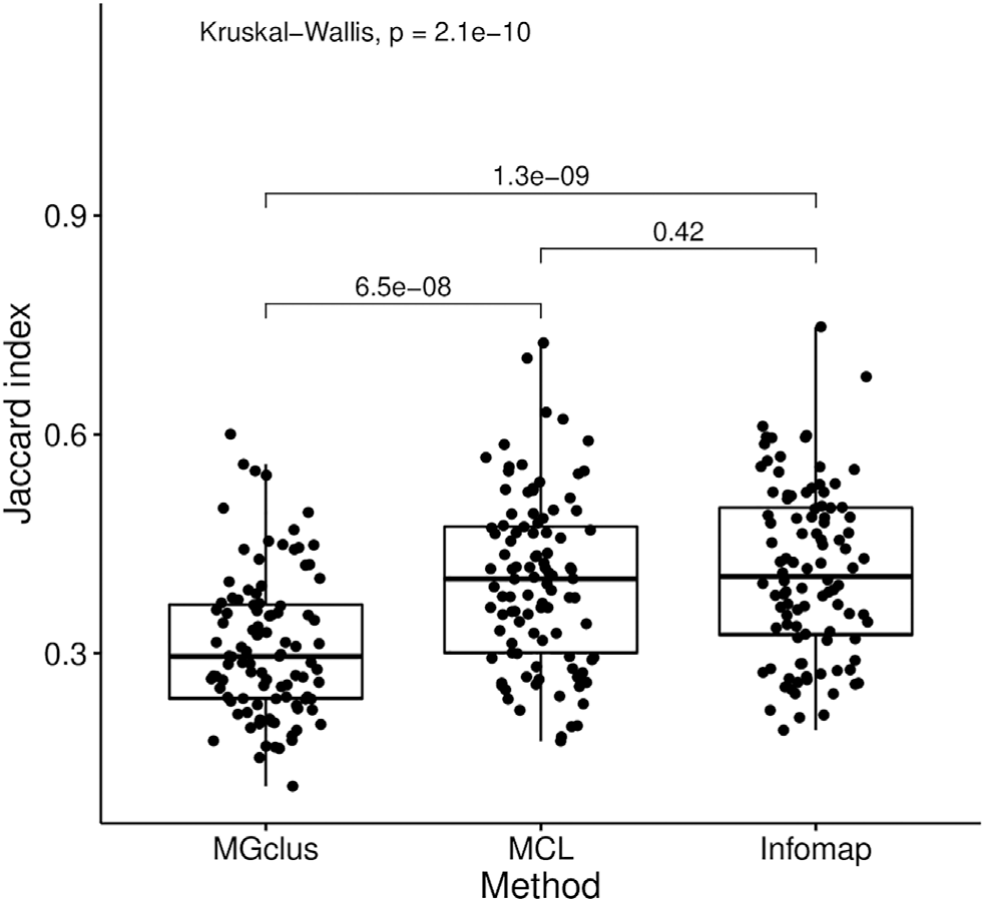
Ability of the clustering methods Infomap, MCL, and MGclus to recover original KEGG pathways in multi-pathway gene sets. Three pathways were grouped into a single gene set prior to clustering. Each module was assigned to the pathway with the greatest overlap, and the Jaccard index of the overlap between true and assigned pathways was computed. The Jaccard index distributions of the clustering methods were compared using Kruskal-Wallis and Wilcoxon tests.

Gene sets derived from experiments typically represent multiple affected pathways. Therefore, mapping these gene sets onto a network such as FunCoup and applying network-based clustering algorithms to divide gene sets into more homogeneous subsets was expected to reduce noise and lead to more accurate pathway analysis. We investigated the effect of clustering on pathway analysis using MGclus, MCL, and Infomap. To assess the clustering performance of these methods on data used in pathway analysis we applied them to gene sets constructed by joining multiple KEGG pathways. Infomap and MCL demonstrated the greatest ability to recover the original pathways with a mean Jaccard index of 41.2% for Infomap and 39.9% for MCL, followed by MGclus at 31% (Figure 3). The difference between Infomap and MCL was not significant (*p* = 0.42), however both Infomap and MCL were significantly different from MGclus, with *p* = 1.3 × $10^{-9}$ and *p* = 6.5 × $10^{-8}$ , respectively.

The original null model of ANUBIX is suitable to capture non-randomness in pathways. However, it may not optimally handle biases present in the query gene set such as genes that are not in the network or genes with very high node degrees. To account for these biases and make the null model more strict we improved the random sampling step to take into account the degree distribution of the query genes. To assess the modified null model generation procedure we created two datasets of random gene sets: one by sampling from the whole genome, and another by sampling from the pool of genes present in the MSigDB CGP gene sets. For the first dataset, both the original and the null model modified ANUBIX had 0% FPR. However, for the second dataset the original ANUBIX had an FPR of 6.6%, while the FPR of the null model modified ANUBIX was only 0.2%.

**FIGURE 4:**
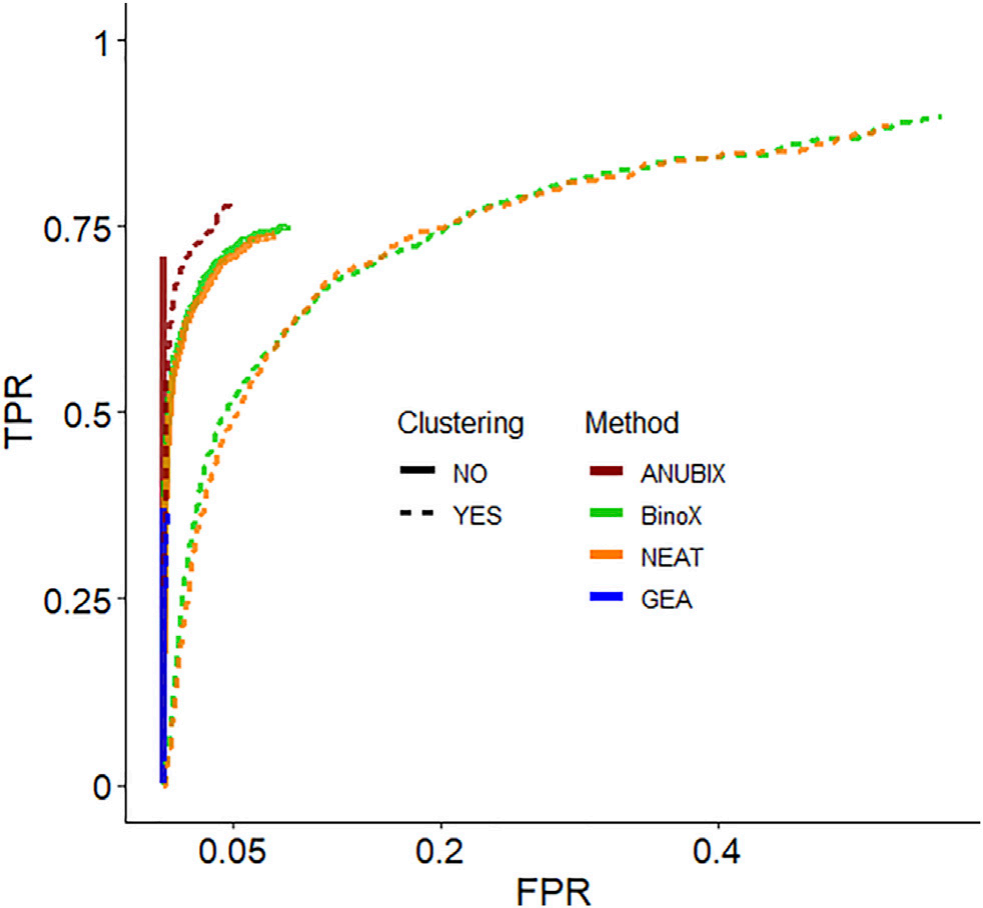
Receiver Operating Characteristic (ROC) curves that measure the performance of each pathway analysis tool, with clustering (dotted lines) and without (solid lines). The used clustering algorithm was Infomap. Only the significantly enriched tests are shown (FDR < 0.05).

We then devised a benchmark to show the effect of pre-clustering of query gene sets. The first part of the benchmark was intended to assess the ability to recover True Positive gene set-pathway pairs. Construction of the benchmark involved bisecting KEGG pathways, merging the first half of several pathways into a heterogeneous gene set and trying to detect enrichment between this gene set and the other bisected halves. In the second part of the benchmark we simulated False Positive gene set-pathway associations by generating random gene sets of the average size of the true positive gene sets. We then assessed the performance of pathway analysis methods: ANUBIX, BinoX, NEAT, and GEA, with, and without pre-clustering on this benchmark. Figure 4 shows the results as a Receiver Operating Characteristic (ROC) curve for MCL and all pathway analysis algorithms. ROC curves when clustering by Infomap and MGclus are in Supplementary Figure S2. The ROC curves only show the statistically significant results at FDR < 0.05, and only for enrichment (i.e. not depletion).

Detailed True Positive Rate (TPR) and False Positive Rate (FPR) results are shown in Table 1. The best balanced performance prior to the application of clustering was demonstrated by ANUBIX, with a TPR of 71% and a FPR of 0%. BinoX and NEAT showed higher TPRs, of 75% and 74% respectively, but had a much higher FPR of 9% and 8%, respectively. As expected, GEA had a low TPR of only 37% due to the low coverage that overlap-based methods tend to have. However, it had a flawless specificity. A significant difference was observed between the results of ANUBIX and the other methods (McNemar´s test, *p* < 0.001).

**TABLE 1:** True positive rate (TPR) and false positive rate (FPR) for combinations of the clustering and pathway enrichment methods run at FDR = 0.05.

	ANUBIX		BinoX		NEAT		GEA	
	TPR	FPR	TPR	FPR	TPR	FPR	TPR	FPR
No clustering	0.71	0.00	0.75	0.09	0.74	0.08	0.37	0.00
MCL	0.73	0.03	0.90	0.57	0.88	0.53	0.35	0.00
MGclus	0.75	0.03	0.88	0.61	0.88	0.56	0.35	0.00
Infomap	0.78	0.05	0.90	0.56	0.88	0.52	0.36	0.00

When applying clustering of the gene sets prior to pathway analysis, we observed a statistically significant (McNemar´s test, *p* < 0.001) increase in TPR for all the network-based pathway enrichment methods ANUBIX, BinoX, and NEAT, but not for GEA, which decreased. The TPR for ANUBIX increased by at most 7 percentage points, when using Infomap, still maintaining an FPR not exceeding the requested FDR level of 5%. BinoX and NEAT exhibited higher increases in TPR of up to 14–15 percentage points. However, this increase came with a very high increase in FPR from 9% to 56–61% for BinoX and from 8% to 52–56% for NEAT. There is a significant difference between the results of the other methods and ANUBIX for all the clustering algorithms (*p* < 0.001).

**FIGURE 5:**
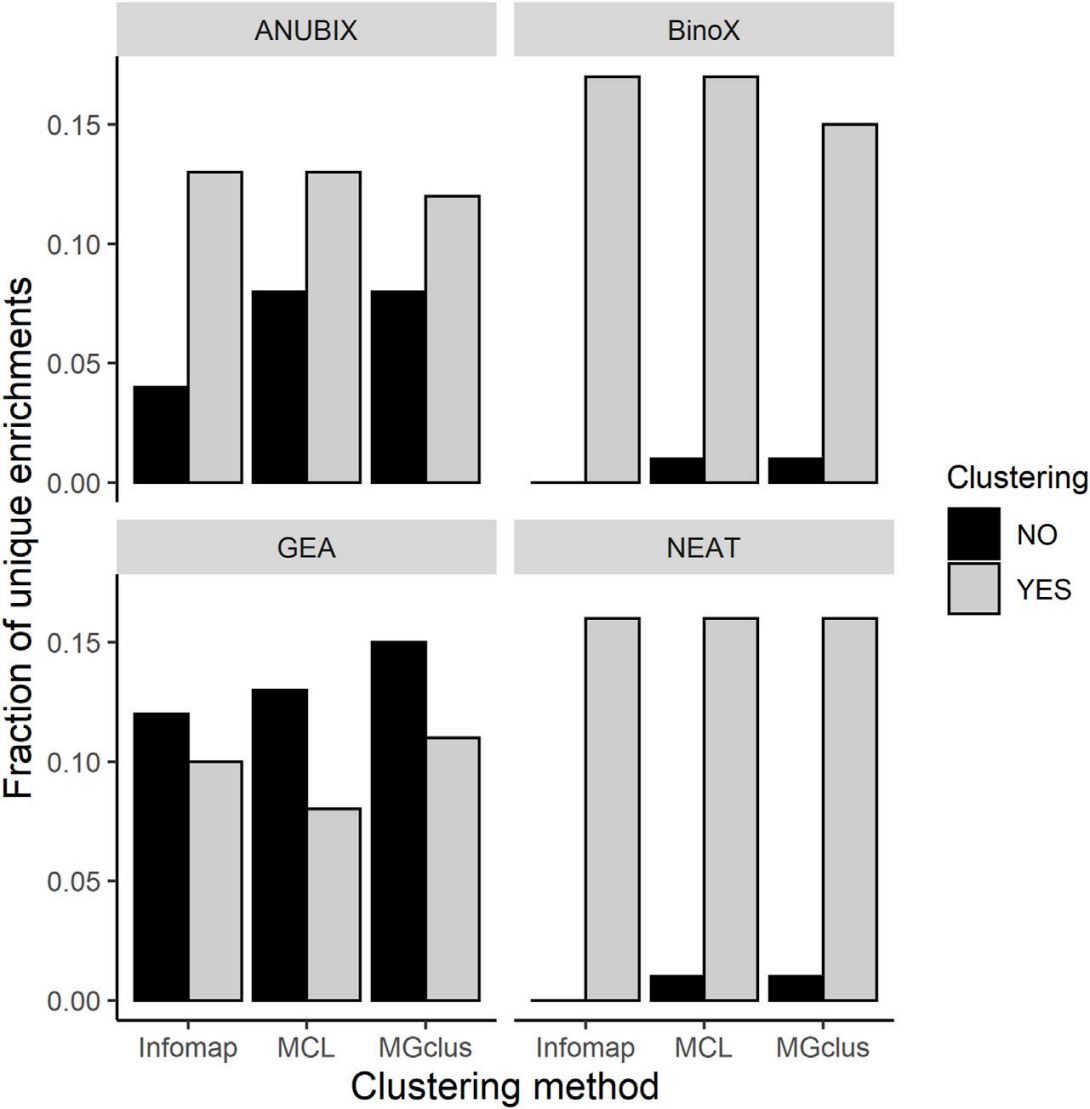
Fractions of unique pathway enrichments found with pre-clustering relative to without pre-clustering, and vice versa, run at FDR = 0.05 for all the combinations of clustering methods and pathway enrichment tools.

We observed that almost all of the enrichments found without clustering were also found using pre-clustering of the query sets (Figure 5). For BinoX and NEAT the fraction of unique enrichments found without clustering were the lowest, below 2%, while for GEA they were the highest at 12–15%. Looking at enrichments only found by pre-clustering, these fractions were generally higher, 8–17%. We further noted that most of the associations, 99.6%, identified by GEA were also found by the network-based methods.

### Clustered Versus Non-Clustered Gene Sets Analysis

A large-scale analysis was carried out for 3302 gene sets from MSigDB/CGP against the 313 human pathways in KEGG, to observe possible benefits of applying clustering to experimental gene sets. Clustering was applied using Infomap and ANUBIX was used for the pathway enrichment analysis. Pathway enrichment analysis web server tools, such as PathBIX (Castresana-Aguirre et al., 2021) or PathwAX (Ogris et al., 2016), are implemented in a way that allows only single gene set queries. By analogy, we studied MSigDB gene sets by assuming independence between gene sets, i.e., multiple testing correction was only performed for the number of pathways each query is compared to.

Clustering of MSigDB gene sets occurred in 2703 of the 3302 gene sets. Pathway analysis without pre-clustering resulted in 129,044 significant (FDR < 0.05) crosstalks across 2,222 gene sets. Clustered analysis produced 122,819 significant crosstalks for 2,178 gene sets, of which 1,890 were shared with the non-clustering approach. The Jaccard index overlap (see Materials and Methods) of significant crosstalks between clustering and non-clustering was 52.5%, and 67.2% of the non-clustering crosstalks were found by the clustering approach while 70.6% of the clustering crosstalks were found by non-clustering.

To show that clustering helps to isolate different mechanisms within a gene set, we used the pathway subclasses as defined in the KEGG database and mapped them to the significant pathway crosstalks from the MSigDB large-scale analysis. Each pathway belongs to a KEGG subclass, and on average 95% of the significant pathways of a certain subclass had crosstalk to just one module in a gene set.

### An Application of Clustered Pathway Enrichment Analysis

To illustrate the usefulness of clustering we provide an example with an MSigDB gene set, HAHTOLA_SEZARY_SYNDROM_UP (Hahtola et al., 2006). More examples can be found in Supplementary File S1 where we provide all significant pathway enrichments found by pre-clustering using ANUBIX and Infomap but not without clustering. The selected example query set contains 99 up-regulated genes (Supplementary Table S1) from peripheral blood samples of Sezary syndrome patients compared to samples from healthy donors. Sezary syndrome is an aggressive form of cutaneous T-cell lymphoma (http://ghr.nlm.nih.gov/condition/sezary-syndrome) and is a rare disease driven by cancerous T-cells with one or several chromosomal abnormalities. We used the web-server PathBIX, which provides both regular ANUBIX and clustered ANUBIX. We ran this gene set against the KEGG pathway database with a FunCoup cutoff of 0.8 and compared the results obtained from non-clustering and clustering. At FDR < 0.05, non-clustering finds 8 significantly enriched pathways, full results in Supplementary Table S2. The top seven pathways belonged to the KEGG classes of “Replication and repair” and “Cell growth and death”, which are pathway classes affected by cancer. The eighth was the “Human T-cell leukemia virus 1 infection” pathway at FDR = 0.01. As opposed to the other seven unspecific cancer related pathways, the last one has been associated with Sezary syndrome (Pancake et al., 1995).

**FIGURE 6:**
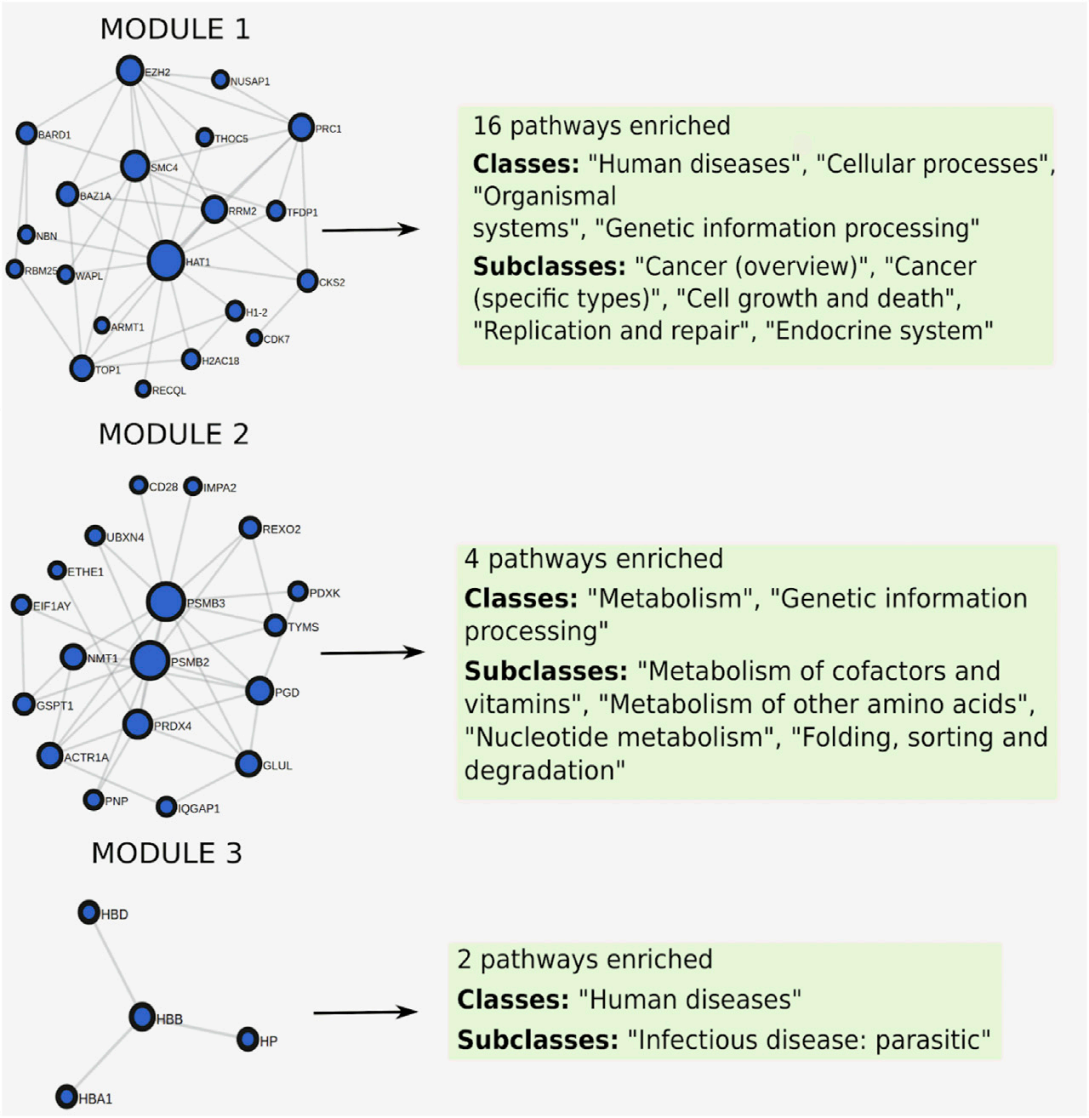
Clustered pathway enrichment analysis of the MSigDB gene set HAHTOLA_SEZARY_SYNDROM_UP. The gene set is divided into 3 modules by applying the network clustering algorithm Infomap. Each module finds different classes of pathways.

**FIGURE 7:**
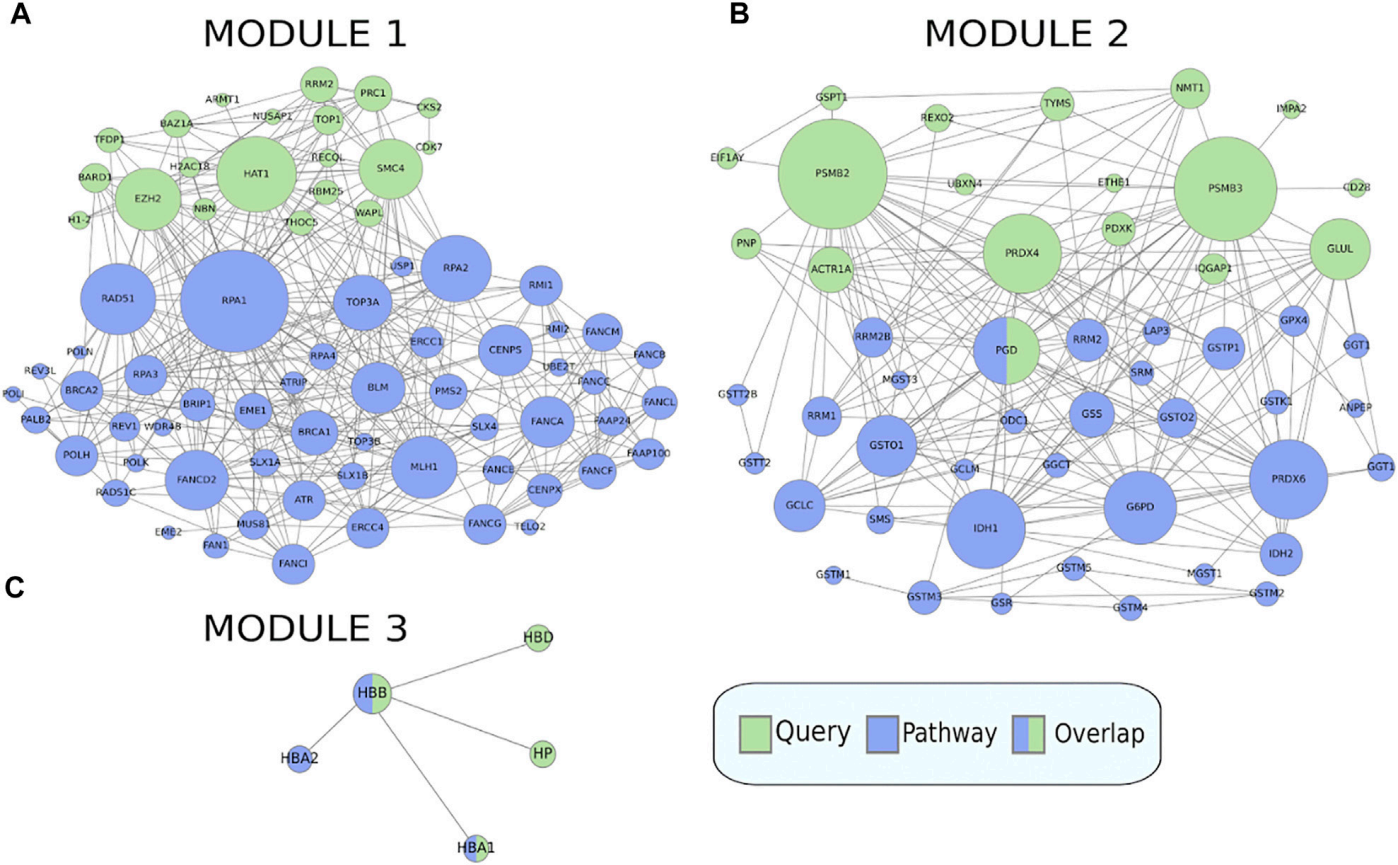
Crosstalk between the three modules in the MSigDB gene set HAHTOLA_SEZARY_SYNDROM_UP and selected KEGG pathways (only genes linked in the network are shown). **(A)** The “Fanconi anemia” pathway which is significantly enriched for crosstalk to module 1. **(B)** The “Glutathione metabolism” pathway which is significantly enriched for crosstalk to module 2. **(C)** The “Malaria” pathway which is significantly enriched for crosstalk to module 3. The query gene set module genes are marked in green and the pathway genes are marked in blue.

When clustering was applied to this gene set, it was split into three modules of size 20, 18, and 4, where each module was enriched for 16, 4, and 2 pathways respectively (Figure 6), full results in Supplementary Table S3. The first module retrieved all the enriched pathways found by the non-clustering approach, while finding additional enriched pathways belonging to the same pathway classes as the pathways found by non-clustering. Pathways relevant to cancer included “Fanconi anemia” (Figure 7A) at FDR = 2.8e−3, a bone marrow failure syndrome whose complications can result in leukemia (Cheung and Taniguchi, 2017), due to a failure in the repair of DNA interstrand crosslinks in the genome (Ceccaldi et al., 2016). The first module was further enriched in other cancer related pathways, such as “Transcriptional misregulation in cancer” at FDR = 1.77e−3. Furthermore, it was enriched in the “Viral carcinogenesis” pathway (FDR = 0.01). This pathway includes genes targeted by the Human T-cell leukemia virus 1 (HTL1 virus), which is thought to be the potential trigger for Sezary syndrome. This is as relevant as the HTL1 infection pathway identified by the non-clustering approach.

The second module finds pathways belonging to the metabolism class, such as “Glutathione metabolism” (Figure 7B) at FDR = 0.02, which is reasonable as glutathione has been proven to effectively block cell death in primary T cells from Sezary patients (Kiessling et al., 2009). Other metabolism pathways like “Purine metabolism” at FDR = 0.03, and “One carbon pool by folate” at FDR = 0.03, are reasonable as purine and folate are potential therapeutic drugs for Sezary syndrome (Oka and Miyagaki, 2019).

The third module finds pathways belonging to the class of parasitic infectious diseases, with “Malaria” at FDR = 3.79e−3 (Figure 7C) and “African trypanosomiasis” at FDR = 8.72e−4. Biomarkers such as miRNA are used for detecting infectious diseases. In malaria, some of the most expressed miRNAs are miR451 and miR92 (Babatunde et al., 2018), where the former is significantly correlated with diagnosis and prognosis of Sezary syndrome, and the latter is downregulated in it (Narducci et al., 2011).

## Discussion

This study aimed at assessing the added benefit of pre-clustering gene sets prior to conducting pathway enrichment analysis. In order to achieve this we evaluated combinations of three network clustering methods in conjunction with one overlap-based and three network-based pathway analysis algorithms. Our findings indicate that pre-clustering increases sensitivity of pathway analysis with network-based methods but observed that it comes with the challenge of risking a high false positive rate. For two of these methods, the improvement in sensitivity came with an unacceptable loss of specificity. However, ANUBIX was able to substantially increase the sensitivity while keeping a high specificity.

The large-scale application of ANUBIX with clustering to the MSigDB gene sets against all KEGG pathways resulted in a similar number of significant enrichments as when no clustering was applied, but about a third of the enrichments were unique to each approach. We further observed that each network module within a gene set tended to be enriched by a different subclass of pathways. This supports the hypothesis that experimentally derived gene sets often represent mixtures of genes with different mechanisms, and isolating these provides a more informative analysis of the different mechanisms that are related to the condition under study. In this analysis we used Infomap for clustering as it was the best method in the benchmarks, and for the pathway enrichment analysis we used ANUBIX since it outperformed the other methods.

Before the pre-clustering analysis, we introduced a modification to the null model of ANUBIX. The new null model of ANUBIX evaluated in the study uses degree-aware sampling of genes in the network instead of randomly sampling genes from the whole genome. This null model modification resulted in a lower FPR compared to the original implementation, hence the modified version of ANUBIX was used in the rest of this study.

A previous benchmark showed that BinoX and NEAT suffer from a relatively high false positive rate (Castresana-Aguirre and Sonnhammer, 2020). To compute the crosstalk between a query gene set and a pathway, BinoX randomizes the network leading to a loss of the internal pathway structure. NEAT does not randomize the network to assess statistical significance but relies on the degrees of the query gene set, pathway, and the whole network, regardless of how that degree is distributed across the pathway. It has been demonstrated that there is a correlation between the FPs of these network-based methods and the fraction of intralinks of the pathways (Castresana-Aguirre and Sonnhammer, 2020), meaning that the less random the pathway topology is, the more prone it is to produce FPs. The distribution of crosstalk between a random gene set and a pathway often suffers from overdispersion, i.e., when the variance is larger than the mean. When this happens, the null distributions of crosstalk assumed by the different methods, binomial (BinoX) or hypergeometric (NEAT), are not appropriate. Both the overdispersion and the high false positive rate are resolved by ANUBIX. Instead of randomizing the whole network which distorts the pathway structure, ANUBIX assesses statistical significance by sampling random gene sets of the same size as the query gene set and computing an expected crosstalk distribution for each pathway. The resulting null distribution is fitted to a beta-binomial distribution, which has been demonstrated to accurately capture overdispersion (Young-Xu and Chan, 2008), and this is used to assess the significance of an observed crosstalk. Even though ANUBIX is the best performing method in that benchmark, we wanted to include other network-based methods to study if clustering could decrease their FPR. However, this issue became even more apparent when clustering was applied. We further observed that the average degree in the unclustered ANUBIX FP gene sets was 82 while the average degree of the genes in FP modules generated from those gene sets increased significantly (*p* < 0.001) to 150, 161, and 193 for Infomap, MCL and MGclus respectively. Statistical significance was assessed using a permutation test by computing the average degree for 2,000 data sets with 100 gene sets in each.

For this benchmark, we did not include quantitative pathway analysis tools, such as GSEA (Subramanian et al., 2005), CAMERA (Wu and Smyth, 2012) or SPIA (Tarca et al., 2009). In order to work, these methods require as input the differential expression of all genes. Several limitations were described previously (Subramanian et al., 2005) when selecting subsets of genes from such a list. Thus, clustering the whole set of genes into independent subsets is unlikely to be beneficial for these methods.

We have demonstrated that the application of clustering of query gene sets prior to pathway analysis improves the sensitivity of all studied pathway enrichment methods, and helps to elucidate complex mechanisms within an experimental gene set. However, pre-clustering is recommended to be used primarily with methods that can control the false positive rate well. The approach finds almost all associations found without clustering, while adding many new ones, and thus represents a powerful new tool in the quest for more accurate pathway analysis.

## Data Availability

The original contributions presented in the study are included in the article/Supplementary Material, further inquiries can be directed to the corresponding author.
