# Supplementary material for: Benefits and Challenges of Pre-clustered Network-Based Pathway Analysis
Source: Front Genet. 2022 May 10;13:855766. doi: 10.3389/fgene.2022.855766 (PMC9127507; doi:10.3389/fgene.2022.855766)
Supplement: Supplementary file 1 [file DataSheet2.PDF]

Benefits and challenges of pre-clustered network-based pathway analysis

Miguel Castresana-Aguirre[1], Dimitri Guala[1], Erik L L Sonnhammer[1*]

[1]Department of Biochemistry and Biophysics, Stockholm University, Science for Life Laboratory, Box 1031, 17121 Solna, Sweden

*To whom correspondence should be addressed.

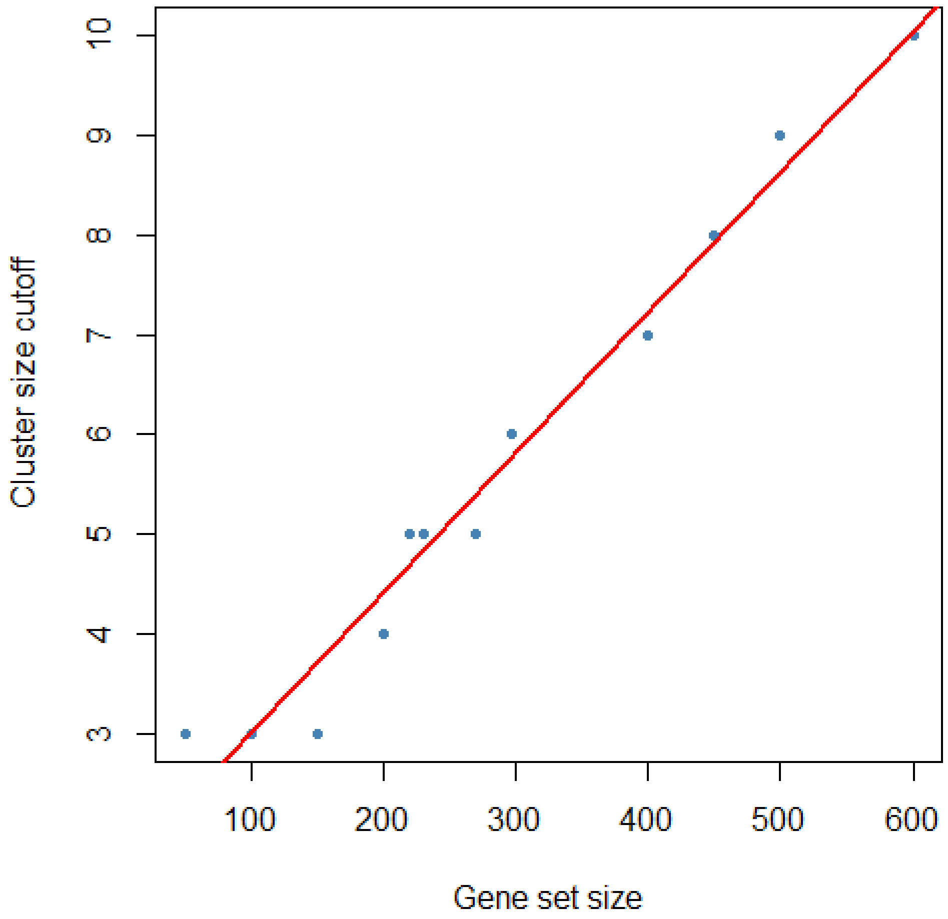

Supplementary Figure 1: Minimum cluster size cutoff required to keep FPR below 0.05 when setting FDR to 0.05, for different gene set sizes.

A

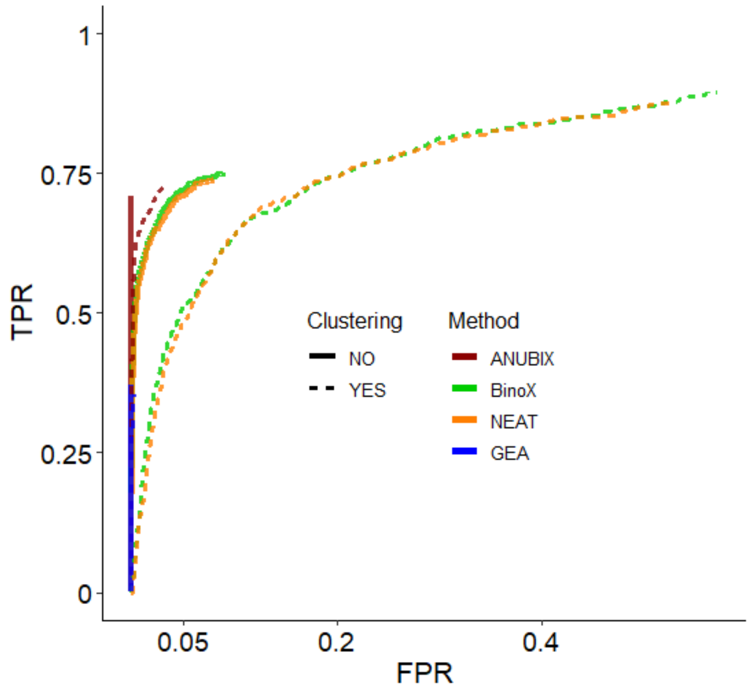

B

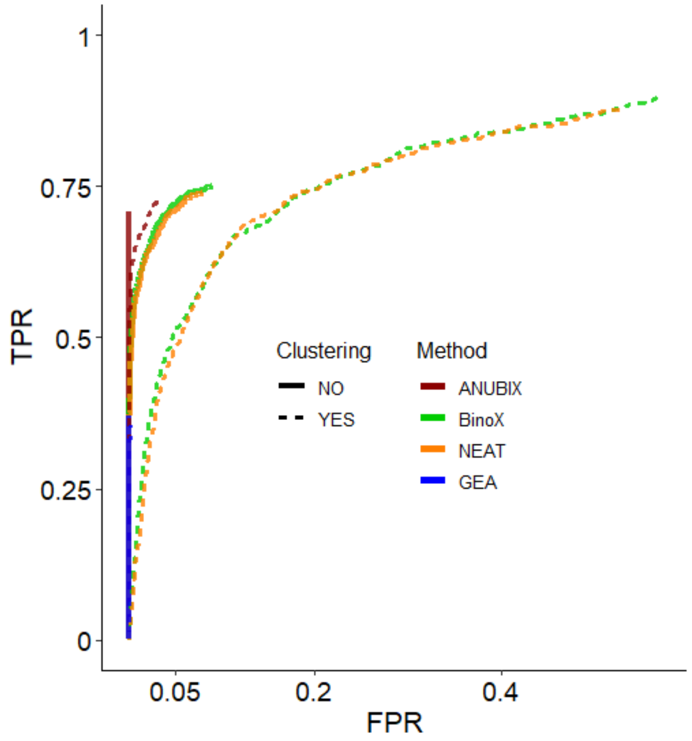

Supplementary Figure 2: Receiver Operating Characteristic (ROC) curves for the performance of each pathway analysis tool on the benchmark, with (dotted lines) and without (solid lines) clustering. Clustering algorithms used were MCL (A), and MGclus (B).

Supplementary Table 1: Genes from the HAHTOLA_SEZARY_SYNDROM_UP gene set.

Genes
ACTR1A, ALAS2, ARMT1, BARD1, BAZ1A, C3AR1, CA1, CCR10, CD28, CD55, CDK7, CES1, CKS2, CRIP1, CSGALNACT1, DYSF, EIF1AY, ETHE1, EZH2, F5, FAR2, FAS, GLIPR1, GLUL, GOLGB1, GPR171, GSPT1, H1-2, H2AC18, HAT1, HBA1, HBB, HBD, HP, ICOS, IDS, IL10RB, IL2RA, IMPA2, IQGAP1, ITM2A, JPT1, KTN1, LGALS8, LILRA5, LIMK2, LPAR6, MEOX1, MMP9, MS4A4A, MTX1, NBN, NCF4, NFE2, NIBAN1, NINJ2, NMT1, NPIPB3, NTAN1, NUSAP1, PAPSS1, PDXK, PGD, PNP, PRC1, PRDX4, PSMB2, PSMB3, PTTG1, RAD51C, RBM25, RECQL, REXO2, RGCC, RNASE2, RNF125, RRM2, RTCA, S100A12, S100P, SAMSN1, SELENBP1, SH3GLB1, SLC25A37, SMC4, SNCA, STAT1, TFDP1, THOC5, TJP2, TLR1, TNFSF10, TOP1, TRAT1, TRIB1, TYMS, UBXN4, WAPL, ZC2HC1A

Supplementary Table 2: Pathway enrichment analysis for the HAHTOLA_SEZARY_SYNDROM_UP gene set without clustering using PathBIX.

pathwayName	p.value	q.value
Base Excision Repair	7.03e-06	1.15e-03
P53 Signaling Pathway	3.57e-06	1.15e-03
Cell Cycle	5.66e-05	6.19e-03
Dna Replication	1.79e-04	1.33e-02
Homologous Recombination	2.02e-04	1.33e-02
Non-Homologous End-Joining	4.43e-04	1.88e-02
Cellular Senescence	3.61e-04	1.88e-02
Human T-Cell Leukemia Virus 1 Infection	4.58e-04	1.88e-02

Supplementary Table 3: Pathway enrichment analysis for the HAHTOLA_SEZARY_SYNDROM_UP gene set with pre-clustering using PathBIX.

pathwayName	Module	p.value	q.value
Cell Cycle	1	1.65e-09	1.61e-06
Base Excision Repair	1	6.47e-09	3.16e-06
Dna Replication	1	3.10e-08	1.01e-05
P53 Signaling Pathway	1	9.50e-08	2.32e-05
Non-Homologous End-Joining	1	3.83e-07	7.48e-05
Homologous Recombination	1	4.73e-07	7.70e-05
Mismatch Repair	1	7.99e-07	1.11e-04
Nucleotide Excision Repair	1	1.75e-06	2.13e-04
African Trypanosomiasis	3	8.04e-06	8.72e-04
Progesterone-Mediated Oocyte Maturation	1	1.17e-05	1.14e-03
Cellular Senescence	1	1.88e-05	1.66e-03
Transcriptional Misregulation In Cancer	1	2.18e-05	1.77e-03
Fanconi Anemia Pathway	1	4.08e-05	2.85e-03
Small Cell Lung Cancer	1	4.09e-05	2.85e-03
Human T-Cell Leukemia Virus 1 Infection	1	5.77e-05	3.75e-03
Oocyte Meiosis	1	6.60e-05	3.79e-03
Malaria	3	6.55e-05	3.79e-03

Viral Carcinogenesis	1	1.88e-04	1.02e-02
Proteasome	2	2.90e-04	1.49e-02
Glutathione Metabolism	2	4.39e-04	2.14e-02
Purine Metabolism	2	7.08e-04	3.25e-02
One Carbon Pool By Folate	2	7.33e-04	3.25e-02
